# *Mycobacterium tuberculosis* MarR family transcription factor Rv0737 regulates bacterial growth and lipid synthesis by targeting the sigL-rslA operon

**DOI:** 10.3389/fmicb.2026.1727573

**Published:** 2026-03-06

**Authors:** Abulimiti Abudukadier, Qiao Zhang, Gang Li, Haiqi Chen, Peibo Li, Zhen Gong, Jianping Xie

**Affiliations:** 1 School of Life Sciences, Institute of Modern Biopharmaceuticals, Southwest University, Chongqing, China; 2 Chongqing Public Health Medical Center, Chongqing, China; 3 Department of Clinical Laboratory, The Second Affiliated Hospital of Anhui Medical University, Hefei, Anhui, China

**Keywords:** cell division/growth regulation, lipid metabolism, MarR transcription factor, *Mycobacterium tuberculosis*, sigL-rslA operon

## Abstract

The MarR family of transcription factors in *Mycobacterium tuberculosis* plays a critical role in bacterial adaptation to host stresses, yet the function of many members remains unknown. Here, we characterize the novel MarR regulator Rv0737 and its homolog Ms_1492 in *M. smegmatis*. Overexpression of Rv0737 severely impaired bacterial growth, cell division, and biofilm formation, while increasing susceptibility to oxidative stress and the cell wall-targeting drug isoniazid. Conversely, deletion of *ms_1492* altered cell envelope permeability and lipid composition, enhanced ATP synthesis, and conferred mild tolerance to H_2_O_2_ and isoniazid. Lipid profiling and transcriptomic analysis revealed significant dysregulation of lipid metabolism genes. Crucially, electrophoretic mobility shift assays demonstrated that both Rv0737 and Ms_1492 directly bind to the promoter region of the sigL-rslA operon, which encodes an alternative sigma factor and its anti-sigma factor. Our findings establish a direct regulatory pathway wherein Rv0737/Ms_1492 modulates bacterial growth, cell envelope integrity, and stress response by targeting the sigL-rslA operon, identifying this system as a potential therapeutic target for combating drug-resistant tuberculosis.

## Introduction

Tuberculosis (TB), caused by the pathogen *Mycobacterium tuberculosis* (*M. tuberculosis*), remains a devastating global health crisis, claiming approximately two million lives annually (WHO, 2024; Osman et al., 2019). Its success as a pathogen is underpinned by a formidable, lipid-rich cell wall intrinsic that provides resistance to antibiotics and host defenses, and by a remarkable ability to adapt to the hostile host environment through sophisticated gene regulation. The emergence of multidrug-resistant (MDR) and extensively drug-resistant (XDR) strains underscores the urgent need to unravel the regulatory networks that govern bacterial adaptation and survival, revealing new vulnerabilities for therapeutic intervention (Merker et al., 2020; Ohji et al., 2020; Veziris et al., 2021).

A cornerstone of *M. tuberculosis* success is its uniquely complex and impermeable cell envelope, rich in idiosyncratic lipids such as mycolic acids, phthiocerol dimycocerosates (PDIMs), and sulfolipids. This structure is not merely a passive barrier but a dynamic interface that actively interacts with the host environment. However, survival within the macrophage—a niche characterized by oxidative and nitrosative stress, nutrient deprivation, acidification, and antimicrobial peptides—demands more than a robust cell wall (Converse et al., 2003; Singh et al., 2018; Rens et al., 2021). It requires the precise, real-time reprogramming of bacterial gene expression. This adaptive capacity is orchestrated by a sophisticated network of transcriptional regulators that sense host-derived stresses and coordinate appropriate physiological responses, encompassing metabolism, repair, and virulence factor production (Flentie et al., 2016).

Among the arsenal of regulatory proteins in *M. tuberculosis*, the MarR (Multiple antibiotic resistance Regulator) family of transcription factors has garnered significant attention for its role in bacterial adaptation and antimicrobial resistance (Will and Fang, 2025). MarR proteins are ubiquitous across bacteria and archaea and typically function as homodimeric repressors. They are characterized by a conserved winged helix-turn-helix (wHTH) DNA-binding domain. A hallmark of many MarR family members is their ability to act as sensors, whose DNA-binding affinity is allosterically modulated by the binding of small-molecule ligands or by post-translational modifications such as the oxidation of conserved cysteine residues, thereby linking gene regulation to metabolic state or oxidative stress (Chauhan et al., 2016; Parish, 2014; Gong et al., 2019; Molle and Kremer, 2010). In *M. tuberculosis*, the MarR family comprises nine members. While the roles of a few, such as Rv0880 (involved in bedaquiline tolerance) (Peterson et al., 2016), Rv1404 (a regulator of acid stress response) (Warrier et al., 2016), and Rv2887 (modulator of drug resistance (Winglee et al., 2015; Warrier et al., 2016) have been elucidated, the biological functions of the majority, including Rv0737, remain completely obscure. This gap in knowledge represents a missed opportunity, as MarR proteins, with their inherent ligand-sensing capabilities, are considered attractive targets for the development of novel anti-infectives that could disrupt pathogenic adaptation.

Here, we focus on Rv0737, a conserved yet uncharacterized putative MarR family protein. Bioinformatic analysis reveals that Rv0737 possesses all the canonical features of the family, including the wHTH domain (Batista et al., 2024). Notably, its amino acid sequence contains conserved cysteine residues at positions often implicated in redox sensing in other MarR proteins, leading us to hypothesize that Rv0737 may function as a redox-sensitive transcriptional regulator (Gong et al., 2019). Furthermore, examination of its genomic context in M. tuberculosis reveals a compelling adjacency: the rv0737 gene is located immediately downstream of the sigL-rslA operon. This operon encodes SigL (σ^54^), an extracytoplasmic function sigma factor, and RslA, its cognate anti-sigma factor (Hahn et al., 2005). The SigL-RslA system is an established regulatory node known to control the expression of genes involved in lipid metabolism, virulence, and stress responses, and it is itself sensitive to redox balance (Dainese et al., 2006). The strategic genomic positioning of rv0737 adjacent to this key operon suggests a potential functional interplay, prompting our secondary hypothesis that Rv0737 may directly regulate the sigL-rslA operon, thereby influencing lipid biosynthesis and stress adaptation pathways. the presence of conserved cysteine residues in Rv0737, analogous to redox-sensing sites in other MarR proteins, suggests it may function as a redox-sensitive transcription factor. This explicitly frames oxidative stress response as a testable possibility derived from sequence analysis, not a premature conclusion.

To experimentally test these hypotheses, we employed the fast-growing, non-pathogenic model organism *Mycobacterium smegmatis* (*M. smegmatis*). This model shares a high degree of genetic and physiological conservation with *M. tuberculosis*, particularly in core processes like cell wall biogenesis and stress response regulation, while offering unparalleled genetic tractability for functional studies. We first investigated the phenotypic consequences of heterologously overexpressing *M. tuberculosis* Rv0737 in *M. smegmatis*. We then constructed a deletion mutant of its direct homolog, *ms_1492*, and performed genetic complementation with *rv0737* to confirm functional conservation. A combination of phenotypic assays, transcriptomics, lipidomics, and *in vitro* biochemistry was used to delineate the function of Rv0737/Ms_1492, identify its direct molecular target, and elucidate its role in governing bacterial growth, cell envelope properties, and susceptibility to host-relevant stresses such as oxidative attack and the first-line drug isoniazid. This work aims to define the role of a neglected MarR family member and uncover a new regulatory pathway with potential implications for combating drug-resistant tuberculosis.

## Materials and methods

### Bacterial strains, media and culture conditions

*M. smegmatis* mc2 155 recombinant strains were cultured in Middlebrook 7H9 broth medium containing 0.05 % (v/v) Tween 80 and 0.2 % (w/v) glycerinum or 7H10 solid medium containing 0.5 % glycerinum, were cultured aerobically without special CO_2_ supplementation. *Escherichia coli* DH5a and BL21 strain was used for gene cloning, cultured in Luria-Bertani (LB) broth or on agar medium. Antibiotics were added when required, ampicillin and hygromycin 100 μg/mL. All bacterial strains were incubated in a rotary (120 rpm) or static incubator at 37 °C (Abo-Kadoum et al., 2021). Growth curves (OD_600_) were monitored by measuring the optical density of diluted cultures using a T6 New Century UV-Vis Spectrophotometer (Beijing Purkinje General Instrument Co., Ltd., China). Static biofilm formation assays were carried out in polystyrene 12-well plates (Corning, Cat# 3513), and biomass was quantified after crystal violet staining.

### Construction of *M. smegmatis* knock-out, complement and *Rv0737* overexpression strains

The Xersite-specific recombination method was used to knockout the *M. smegmatis Ms_1492* gene. First, we constructed gene fragments A and B containing homology arms of the target fragment. The two genes were used as primers and templates to amplify each other by PCR to obtain the AB fragment and connect the T carrier to obtain the recombinant plasmid C for storage. The hygromycin gene fragment was connected to the recombinant T vector to obtain the recombinant plasmid D. The recombinant plasmid D was transformed into smegmatis recipient bacteria (containing pJV53 plasmid) to verify the transformation. Homologous fragments recombine with target gene fragments, and the expression of recombinase induces homologous recombination. Bacterial liquid PCR verification. The positive colony was the unmarked *Ms_1492* deletion mutant (Δ*Ms_1492*) that was passaged 10 times in 7H9 liquid medium without hygromycin and finally lost the Hyg resistance cassette (Yan et al., 2021; Ling et al., 2021).

To generate the complemented strain, the *rv0737* gene was amplified from *M. tuberculosis* H37Rv genomic DNA using primers listed in Supplementary Table 1. The PCR product was cloned into the EcoRI and BamHI sites of the *E. coli*-mycobacterial shuttle vector pNIT. This vector contains a nitrile-inducible promoter (Pnit), a kanamycin resistance marker and a Myc tag. The resulting plasmid, designated pNIT_Rv0737, was verified by Sanger sequencing and then electroporated into the previously constructed *M. smegmatis* wild type strain and Δ*Ms_1492* mutant strain. Transformants were selected on Middlebrook 7H10 agar plates containing 50 μg/mL kanamycin, yielding the complemented strain Δ*Ms_1492*::pNIT_*rv0737* and overexpression strain Ms_pNIT_*rv0737.* For experiments requiring gene induction, cultures were supplemented with 0.2% (v/v) ε-caprolactam (CPL).

### Time-kill assays and survival assay

For spot assays, mid-log phase cultures were adjusted to OD_600_ = 0.8, serially diluted, and spotted onto 7H10 agar plates containing isoniazid (0.32 μg/mL). Plates were incubated at 37 °C for 3–5 days. Aliquots were taken at intervals, serially diluted, plated, and colonies were counted after 48 h to determine viable counts. First convert the CFU value to Log_10_ (CFU/mL), then compare the Log_10_ (CFU/mL) of the treatment group with that of the control group.

### Electrophoretic mobility shift assay

The promoter region of the sigL-rslA operon (≈280 bp) was amplified by PCR. The *rv0737* and *Ms_1492* genes were heterologously expressed in *E. coli* BL21 (DE3) and the recombinant proteins were purified via nickel-affinity chromatography. For binding reactions, 100 ng of promoter DNA was incubated with purified protein (0–6 μM) in EMSA buffer [10 mM Tris-HCl pH 7.5, 50 mM KCl, 1 mM DTT, 5% glycerol, 0.05% NP-40, 50 μg/mL poly(dI-dC)] for 30 min at 37 °C. Bovine serum albumin (BSA, 6 μM) was included as a non-specific protein control, and promoter fragments of Ms_1488 and Ms_1494 were used as non-target DNA controls. Protein-DNA complexes were resolved on a 6% non-denaturing polyacrylamide gel in 0.5x TBE buffer at 100 V for approximately 90 min at 4 °C. Gels were stained with SYBR™ Green I nucleic acid stain and visualized under UV illumination.(Yan et al., 2021). Protein-DNA complexes were resolved on 6% native polyacrylamide gels prepared with a Mini-PROTEAN Tetra Cell system (Bio-Rad, USA), run in 0.5x TBE buffer at 100 V for 90 min at 4 °C, and stained with SYBR™ Green I Nucleic Acid Gel Stain (Invitrogen, Cat# S7563).

### Detection of cell membrane permeability

*M. smegmatis* was cultured to OD_600_ 0.8, and the bacteria were collected by centrifugation at 8,000 rpm for 10 min. Adjust the OD_600_ 0.8, pipette 200 μL of bacterial solution into a 96-well microtiter plate, and quickly add Ethidium Bromide (EB) and Nile red (NR) with a final concentration of 10 μg/mL. After mixing, use a multi-function microplate reader to detect the fluorescence value of the sample. Three replicates were set for each sample, and the fluorescence data were recorded every 5 min, and the experiment lasted for 1 h. The accumulation of the two dyes was determined by calculating the fluorescence using Microplate Reader with an excitation of 540 nm and an emission of 590 nm (Abo-Kadoum et al., 2021).

### Determination of ROS content and ATP content in bacteria

The WT, Δ*Ms_1492* and Δ*Ms_1492*::pNIT_*Rv0737* strains were centrifuged at 8,000 × *g* 10 min, the pellet was washed with 1 × PBS three times, the OD_600_ was adjusted to about 0.4 and bacteria ROS content were measured with the ROS Assay kit (Beyotime, Shanghai, China). The WT, Δ*Ms_1492* and Δ*Ms_1492*::pNIT_*Rv0737* strains bacteria ATP content were measured with the ATP Assay kit (Beyotime, Shanghai, China) and the multifunctional microplate reader (Tecan, Männedorf, Switzerland).

### H_2_O_2_ sensitivity assays

Mid-log phase cultures were diluted and exposed to 2 mM H_2_O_2_ in 7H9 medium. Growth (OD_600_) was monitored over 2 h. A failure to increase OD under these conditions is interpreted as growth inhibition due to stress sensitivity (Parish and Roberts, 2015).

### Analysis of lipid components in the cell wall of *M. smegmatis*

*M. smegmatis* cells were harvested for lipid extraction during the mid-logarithmic growth phase at an OD_600_ of 0.8 ± 0.1, ensuring a consistent physiological state relevant to cell wall biosynthesis. Total lipids were extracted from cell pellets using a modified Folch method (Slayden and Barry, 2001). Resuspend the cells with 100 mL methanol: chloroform: 0.3% NaCl (9:10:3, v/v/v) and stir for 4 h. The solution was filtered through a magnesium silicate column to remove insoluble impurities, and the filtrate was mixed with 30 mL of chloroform: 0.3% NaCl (1:1, v/v) and stirred for 1 h. After the solution is allowed to stand for stratification, the lower liquid phase is sucked and dried to obtain the bacterial cell wall lipid extract. Resuspend the cells with 20 mL methanol: 0.3% NaCl (10:1, v/v), add 10 mL petroleum ether, and stir for 15 min. After the solution was left to stand for stratification, the upper layer mixture was sucked and dried to obtain a non-polar lipid extract. A total of 17.3 mL of methanol-chloroform-0.3% NaCl (10:9:3, v/v/v) was added to the lower aqueous phase and stirred for 1 h. The solution was allowed to stand for stratification, and the lower liquid phase was recovered and dried to obtain the bacterial cell wall polar lipid extract. An appropriate amount of the extracted cell wall lipids was pipetted and dropped onto a silica gel plate with a capillary. After drying the silica gel plate, lipid separation experiments were carried out in different mobile phases.

For comparative analysis via thin-layer chromatography (TLC) and subsequent profiling, equal amounts of total lipid extract – normalized by the wet weight of the cell pellet prior to extraction – were loaded onto each lane. This wet-weight normalization serves as a robust and standard loading control, directly correlating the loaded lipid material with the original biological biomass and thereby enabling accurate comparison of lipid composition between strains. Thin-layer chromatography (TLC) was performed on Silica Gel 60 F_254_ plates (Merck, Cat# 1.05715). For all thin-layer chromatography (TLC) analyses, lipid extracts were normalized prior to loading based on the wet weight of the cell pellet from which they were derived. This is a standard and rigorous method to ensure equal representation of biomass across samples. Specifically, lipids were extracted from equivalent amounts of cell material (by wet weight), and equal volumes of these standardized extracts were applied to the TLC plate.

### RNA extraction and RT-qPCR

After cultivating WT and Δ*Ms_1492* with 100 mL of 7H9 liquid medium to the log phase, the cells were collected by centrifugation at 8,000 rpm and washed twice with 1 × PBS for bacterial RNA extraction and RT-qPCR experiments. RNA extraction was done following the manufacturer’s instructions (TIANGEN, China). Three replicates and set a negative control (without template) were set for each sample, which were then amplified using the Bio-Rad PTC-200 fluorescence quantitative PCR instrument (Yan et al., 2021).

### Metabonomics measurement and analysis

The WT, Δ*Ms_1492* and Δ*Ms_1492*::pNIT_*Rv0737* strains were cultured in 300 mL 7H9 liquid medium to the logarithmic growth phase (OD_600_ = 0.8). The cell pellet was collected by centrifugation and washed once with 1 × PBS. The pellets were air dried, and liquid nitrogen was used to quick-freeze the samples and placed in −80 °C for storage. The bacterial samples were sent to the company for non-targeted metabolome assay. The methodology followed a previously described method (Yan et al., 2021).

### Reagents and commercial kits

All chemical reagents and commercial kits used in this study were sourced as follows. Culture media components: Tryptone and yeast extract were from OXOID; Difco™ Middlebrook 7H9 and 7H10 broth were from BD Biosciences; sodium chloride, Tween 80, and agar were from Sigma-Aldrich; glycerol was from Chongqing Chuandong Chemical (Group) Co., Ltd. Antibiotics and dyes: Kanamycin, ampicillin, and isoniazid were from Sangon Biotech (Shanghai); hydrogen peroxide (35%) was from Aladdin Biochemical Technology; hygromycin, EB, and NR were from Dingguo Changsheng Biotechnology (Beijing). Molecular cloning: T4 DNA ligase, Taq DNA polymerase, restriction enzymes, dNTPs, and related buffers were from TaKaRa; DNA markers (DL2000, DL15000), PCR purification kits, plasmid extraction kits (Axygen), and genomic DNA extraction kits were used. Agarose and SDS-PAGE: Agarose, SDS, APS, and TEMED were from Sangon Biotech (Shanghai). RNA and qRT-PCR: TRIzol reagent was from Invitrogen; chloroform, isopropanol, and ethanol were from Titan Chemical; RNA purification kits were from TIANGEN Biotech (Beijing); reverse transcription and quantitative PCR kits were from TaKaRa. Biochemical assays: ROS Assay Kit and ATP Assay Kit were from Beyotime Biotechnology (Shanghai). Western blotting: Mouse anti-Myc and anti-His primary antibodies, and goat anti-mouse IgG-HRP secondary antibody were from Baoguang Biotechnology (Chongqing). Lipid extraction: Chloroform, petroleum ether, ethyl acetate, methanol, and acetone were from Titan Chemical.

### Statistical analysis

Three independent biological replicates were performed for each experiment. A biological replicate is defined as an experiment started from separate, freshly inoculated cultures grown on different time point. The data were analyzed using student’s two-tailed *t*-test. Significance was defined as *P*-value (*P < 0.05; **P < 0.01; ***P < 0.001; ns, not significant). Error bars represented standard deviation (SD).

## Results

### The conservation of the MarR family gene *rv0737* and its homologous genes in the genus *Mycobacterium* is relatively high

**FIGURE 1:**
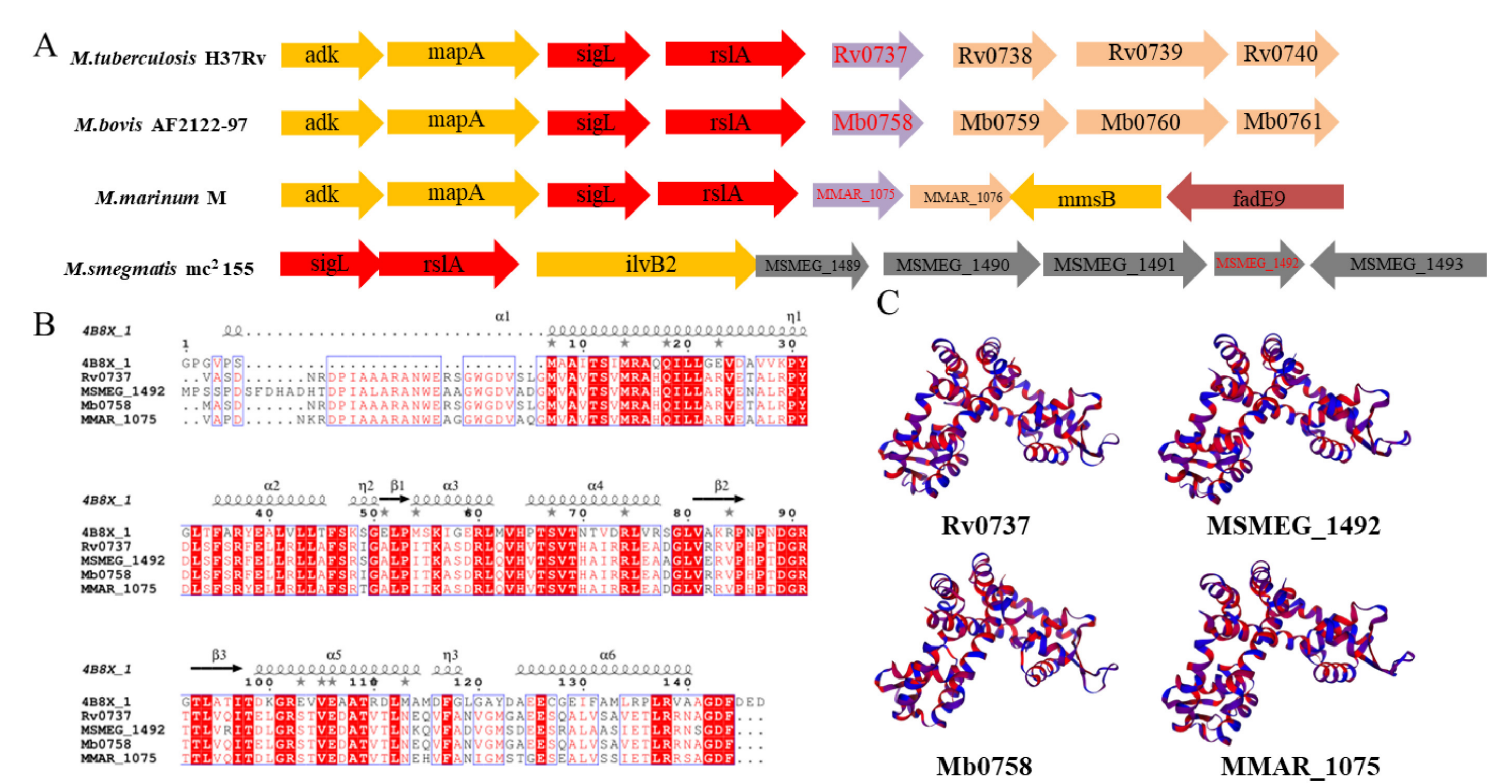
Conservation analysis of Rv0737. **(A)** Genomic context of the sigL-rslA operon (red) and neighboring genes in different mycobacterial species. The position of the Rv0737 ortholog (purple, regulatory protein) is variable, indicating genomic rearrangement. The functional ortholog in *M. smegmatis* (Ms_1492) is located within a separate gene cluster. **(B)** Conservation analysis of the amino acid sequence of Rv0737, red indicates that the residues are highly conserved. **(C)** Tertiary structure of the Rv0737 homologs.

Bioinformatic analysis revealed that Rv0737, a putative MarR-family transcriptional regulator, is highly conserved among pathogenic and non-pathogenic mycobacteria. Multiple sequence alignment of homologous proteins highlighted conserved functional domains, including redox-sensing cysteine residues and DNA-binding motifs (Figure 1). This high degree of conservation permitted the use of *M. smegmatis* as a surrogate model for functional studies. Furthermore, the conservation of cysteine residues within the Rv0737/Ms_1492 protein sequence (Figures 1B, C) presents an intriguing possibility that its DNA-binding activity or regulatory function could be modulated by redox status, a common feature among MarR-family proteins involved in stress sensing.

### *Rv0737* overexpression impairs growth and cell division in *M. smegmatis*

**FIGURE 2:**
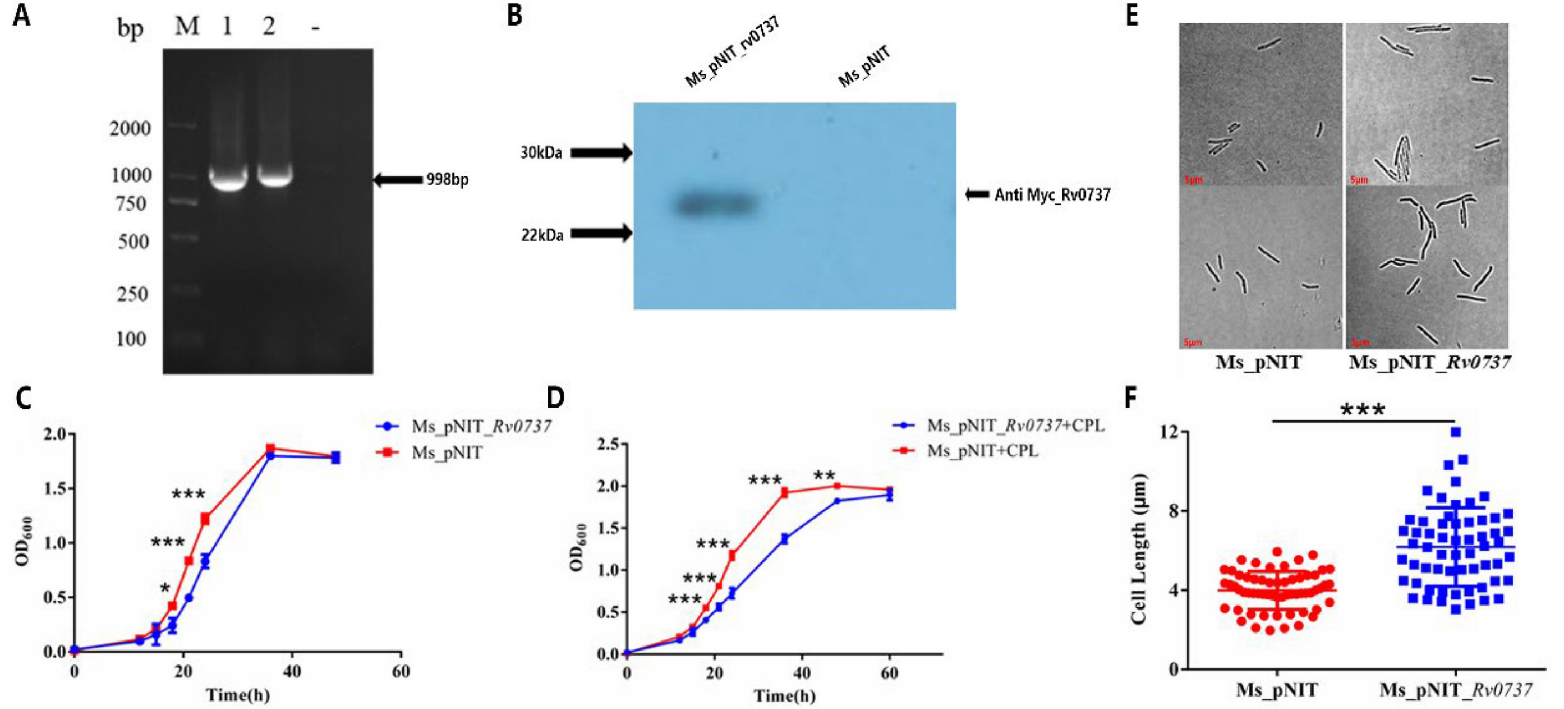
**(A)** PCR validation of *M. smegmatis* overexpression strain Ms_pNIT_*Rv0737*. M: Marker, A: 1-3 are PCR amplification products of *rv0737*, B: 1-2 are PCR amplification products of *rv0737*, “-” is a negative control. **(B)** WB validation of *M. smegmatis* overexpression strain Ms_pNIT_*Rv0737*. **(C)** The growth curve of the overexpression strain measured without CPL. **(D)** The growth curve of the overexpression strain measured when CPL was added. CPL: inducer caprolactam. **(E)** Cell lengths of overexpressing strains and empty strains in 7H9 medium containing Tween 80. **(F)** Statistical scatter plot of cell length of overexpression strains and empty strains. Symbols are: *p < 0.05, **p 0.01, ***p 0.001.

In order to verify whether *rv0737* overexpression affects the normal growth of *Mycobacterium smegmatis*, we constructed Ms_pNIT_*rv0737* with pNIT plasmid containing the Myc tag, and verified by PCR and WB (Figures 2A, B and Supplementary Figure 1). We measured the growth curves of the overexpression strain Ms_pNIT_*rv0737* and the control empty strain Ms_pNIT. The detection results showed that the growth rate of the overexpression strain was significantly slowed down in the early and middle stages, and the growth delay rate was about 9 h lower than that of the empty strain (Figures 2C, D). Ms_pNIT_*rv0737* strain will add caprolactam (CPL) as an inducer to induce high gene expression. In order to detect the effect of pNIT_*rv0737* recombinant plasmid on Ms_pNIT_*rv0737* strain when it is not induced to have low expression, the same method was used again to determine the growth curve of *M. smegmatis* without caprolactam. The results showed that although the growth retardation effect of ms_pnit_rv0737 strain was weakened without caprolactam, there was still a 3H growth delay (Figures 2C, D). Since overexpression of *rv0737* was observed to delay the growth of *M. smegmatis*, we further examined whether it affected bacterial cell division. The cell lengths of the overexpression strain and the empty-vector control strain were measured. The average length of the overexpression strain was 6.196 μm, compared with 4.090 μm for the control strain (Figure 2E).

### Rv0737 disrupts biofilm formation and enhances oxidative stress sensitivity

**FIGURE 3:**
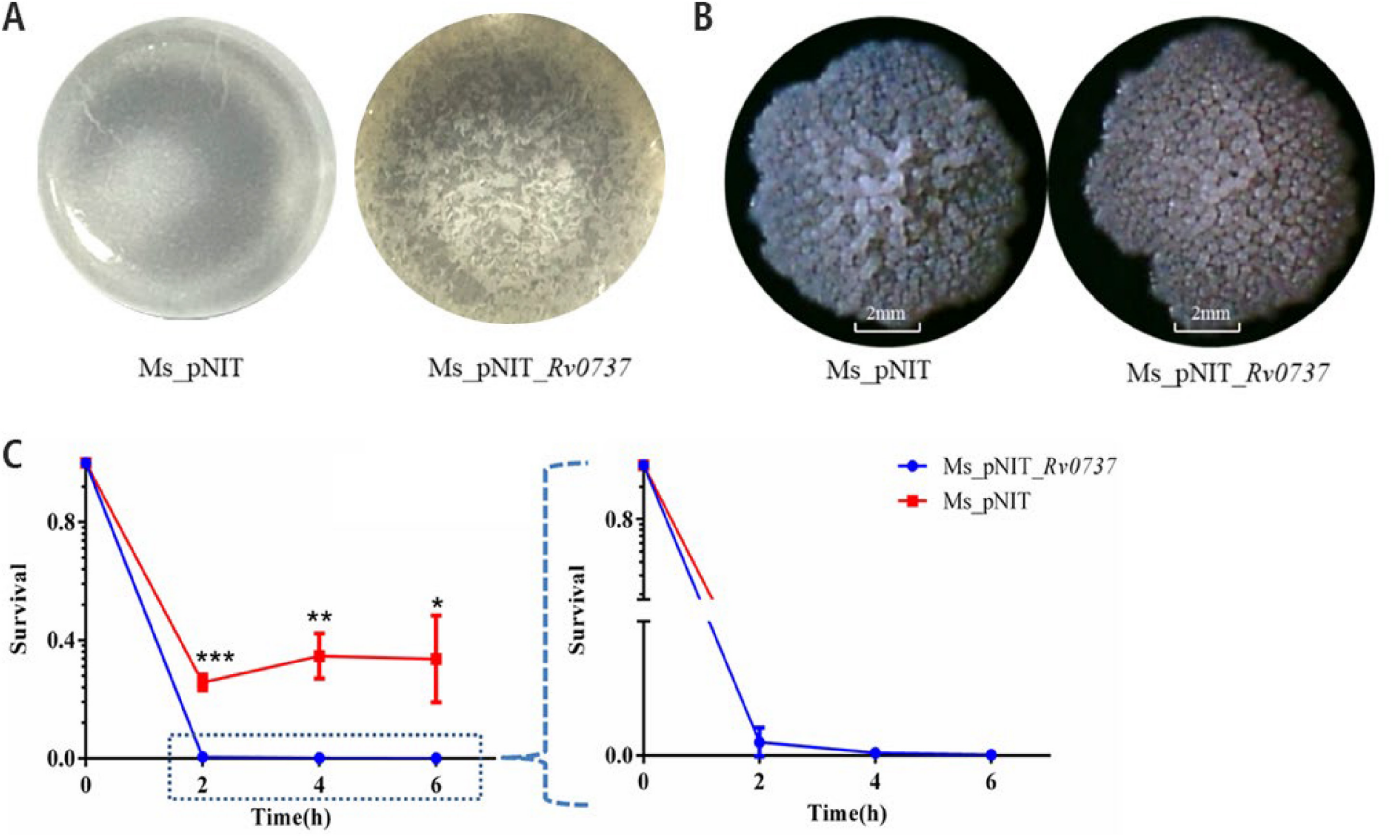
**(A)** Biofilm-forming ability of *M. smegmatis* overexpression strains versus empty strains. **(B)** Single colony morphology of overexpression strain and empty strain on 7H10 medium. **(C)** Overexpression of *rv0737* affects *M. smegmatis* oxidative stress response. Symbols are: *p < 0.05, **p 0.01, ***p 0.001.

Beyond growth defects, Rv0737-overexpressing cells exhibited severely compromised biofilm architecture, showing fragmented and unstructured mats compared to the highly wrinkled and coherent biofilms of the control strain (Figure 3A). Colony morphology on solid media remained unchanged (Figure 3B). Notably, the overexpression strain displayed significantly enhanced sensitivity to hydrogen peroxide (2 mM), indicating a critical role for Rv0737 in regulating the oxidative stress response (Figure 3C).

### Rv0737/Ms_1492 functions as a pro-oxidant cellular factor whose overexpression is detrimental to mycobacterial fitness

**FIGURE 4:**
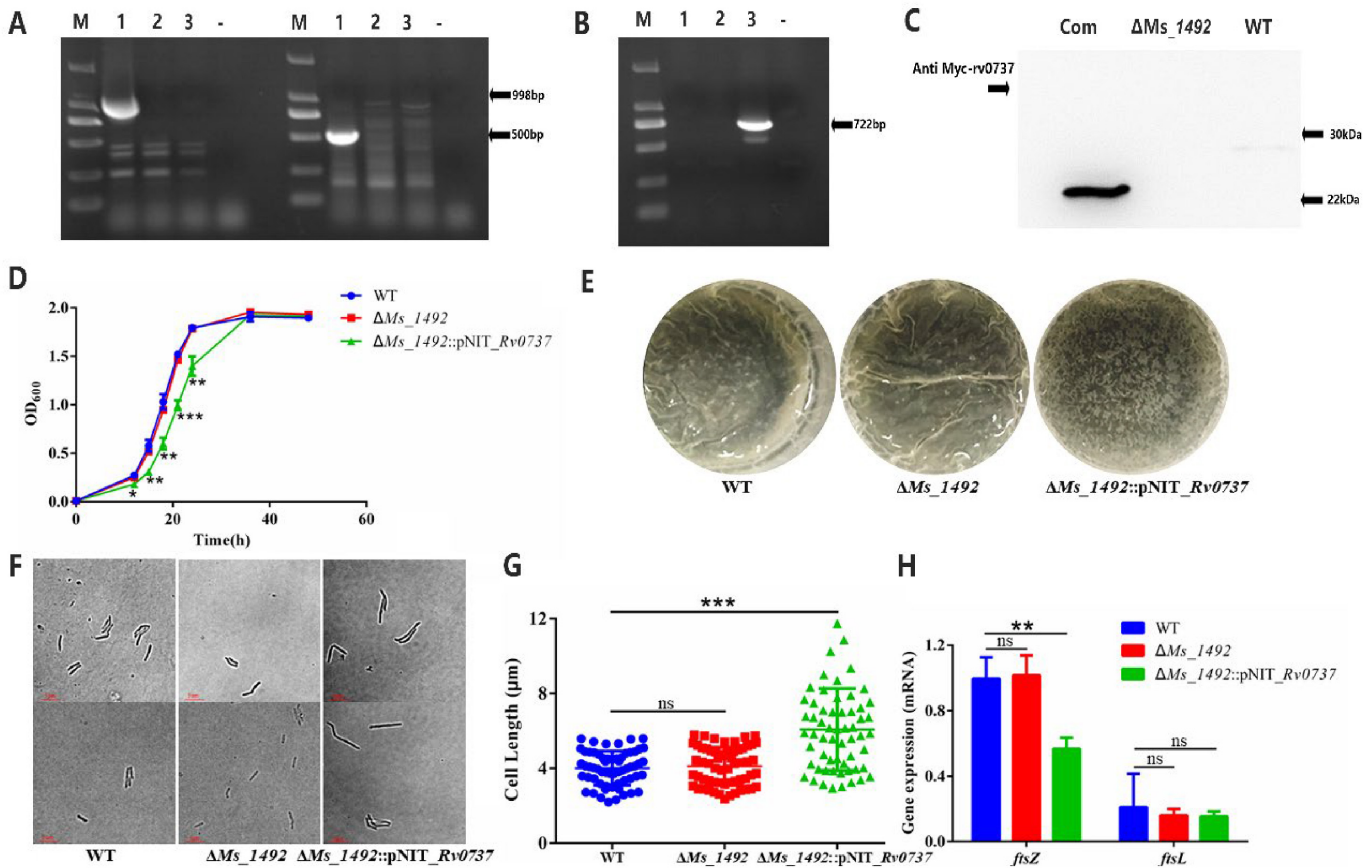
**(A,B)** PCR validation of wild-type strain (left), deletion strain (right). and complemented strains **(B)**. M: Marker, primers used for A and B: 1–3 were qc-validated -F/R, hyg-validated F/R, pNIT universal F/R, “-” was a negative control. **(C)** WB validation of wild-type strains (WT), deletion strains (Δ*Ms_1492*) and complemented strains (Com). **(D)** Growth curves of wild-type strains, deletion strains and complemented strains. **(E)** Biofilm-forming ability of wild-type strains, deletion strains and complemented strains. **(F,G)** Cell lengths of wild-type strains, deletion strains and complemented strains in 7H9 medium containing Tween 80, as well as scatter plots of cell length statistics. **(H)** The mRNA level of *ftsZ* and *ftsL*. Error bars indicate the standard deviation (SD) and the statistics was calculated using a Student’s unpaired two-tailed *t*-test by the GraphPad Prism software. Symbols are: *^ns^p* > 0.05; *p < 0.05, **p ≤ 0.01, ***p ≤ 0.001.

To delineate the native function of Rv0737, its direct homolog in *M. smegmatis*, *Ms_1492* was deleted via homologous recombination (Δ*Ms_1492*). The mutant was subsequently complemented with the *rv0737* gene (Δ*Ms_1492*::pNIT_*Rv0737*). Genotypic validation was confirmed by PCR and phenotypic rescue via Western blot (Figures 4A–C and Supplementary Figures 2–4, 8).

*In vitro* growth kinetics and bacterial length of both wild type (WT) and *Ms_1492* deletion strain (Δ*Ms_1492*) in *M. smegmatis* showed no significant difference (*P* > 0.05) when grown in 7H9 medium. But the growth of the complemented strain was slowed down, showing 3 h growth delay relative to the WT strain (Figure 4D). The biofilm difference between WT and Δ*Ms_1492* strain was similar (Figure 4E). Complemented strain (Δ*Ms_1492*::pNIT_*Rv0737*) bacterial length was significantly longer (Figures 4F, G). And by RT-qPCR experiments, it was found that the complemented strain mRNA level of *ftsZ*, a pivotal cell division protein, was lower than WT and deletion strains (Figure 4H). Furthermore, cell division was markedly impaired in the Rv0737-overexpressing strain, as evidenced by a significant increase in average cell length compared to the empty vector control (3.796 vs. 2.490 μm; Figure 2F). This morphological alteration was associated with a concomitant decrease in the mRNA level of ftsZ, which encodes an essential division protein (Figure 4H).

### Rv0737/Ms_1492 modulates cell envelope permeability and lipid composition

**FIGURE 5:**
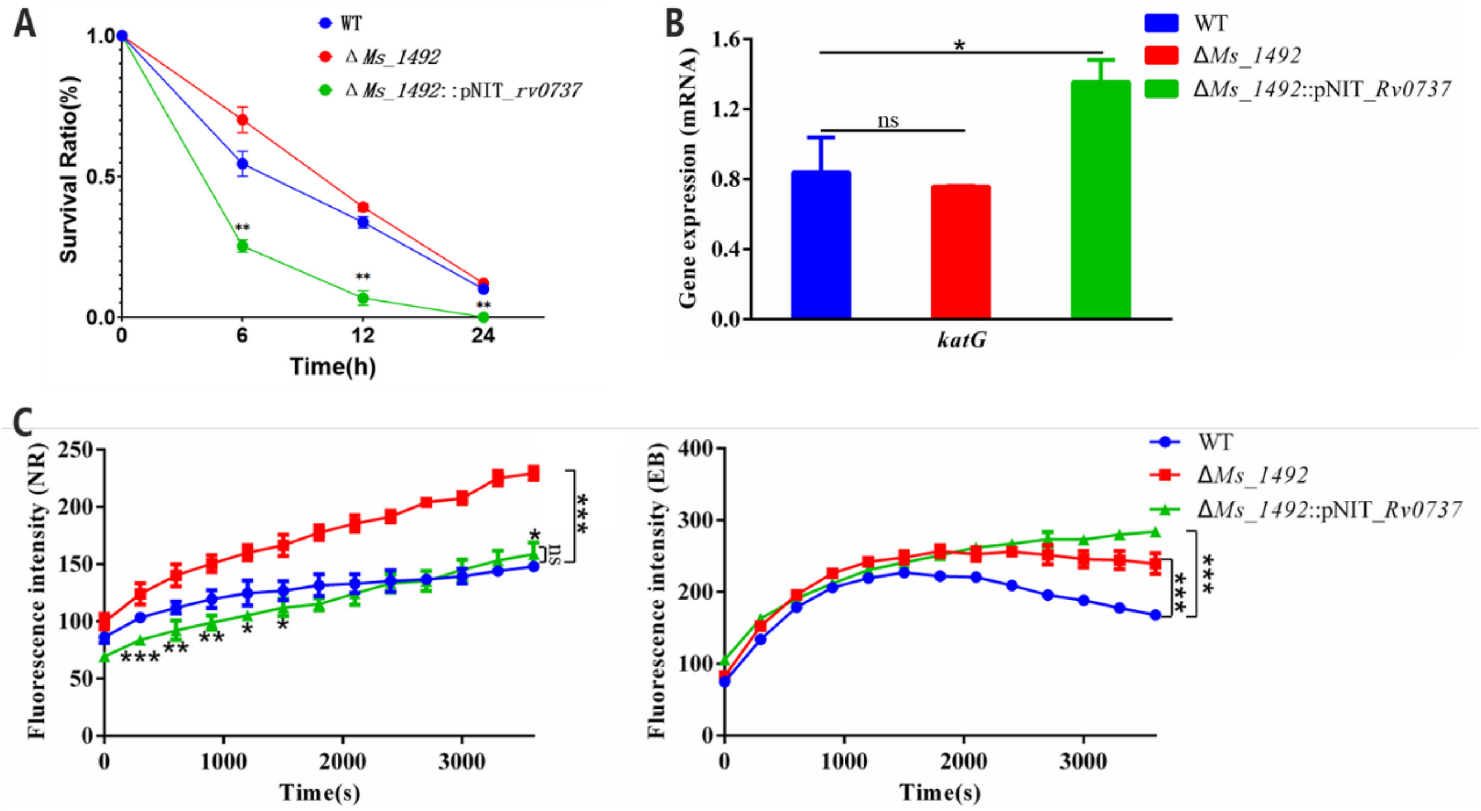
**(A)** Deletion of *Ms_1492* enhances isoniazid tolerance in *M. smegmatis*. Time-kill assays demonstrating that WT, Δ*Ms_1492* and Δ*Ms_1492*::pNIT_*rv0737* strains were performed colony forming unit (CFU) on 7H10 plate with isoniazid (0.36 mg/mL). **(B)** Transcription of *katG* was analyzed using RT-qPCR in the *M. smegmatis* WT, Δ*Ms_1492* and Δ*Ms_1492*::pNIT_*Rv0737* strains. **(C)** Intracellular accumulation of NR (left) and EB (right) in wild-type strains, deletion strains, and apoplectic strains. Error bars indicate the standard deviation (SD) and the statistics was calculated using a student’s unpaired two-tailed *t*-test by the GraphPad Prism software. Symbols are: *^ns^p* > 0.05; * *p* < 0.05, ** *p* ≤ 0.01, ***p ≤ 0.001.

Rv0737 was shown to regulate oxidative stress defense and ability of forming biofilm. Isoniazid (INH) is the first-line anti-tuberculosis drug for the prevention and treatment of tuberculosis. This prodrug is activated by the catalase-peroxidase KatG in *M. tuberculosis*, thereby inhibiting the synthesis of mycolic acids required for the mycobacterial cell wall (Ascenzi et al., 2013; Unissa et al., 2016). In addition, the activation of isoniazid by KatG also produces some free radical species (such as NO) with anti-mycobacterial activity (Timmins et al., 2004). Thus, we analyzed the phenotypes of the WT, Δ*Ms_1492* and Δ*Ms_1492*::pNIT_*Rv0737* strains under 10 × MIC (0.036 mg/mL) INH treatments. Δ*Ms_1492* strains was more tolerant to INH stress in comparison to the WT strain in survival assays (Figure 5A),and RT-qPCR results showed that transcription of katG was up-regulating in Δ*Ms_1492*::pNIT_*Rv0737* strains which was sensitive to INH (Figure 5B). We did not observe significant katG downregulation in the Δ*Ms_1492* strain itself (Figure 5B), ruling this out as a primary mechanism. The downregulation in the complemented, INH-sensitive strain further supports the inverse relationship between katG levels and INH sensitivity in this system.

In addition, SigL had demonstrated to modulate cell wall biosynthesis via up-regulating the expression of polyketide synthases such as pks7/pks10 and Rv1833c (Hahn et al., 2005). We measured the accumulations of lipophilic compound Nile Red (NR) and hydrophilic Ethidium Bromide (EB) compound in WT, Δ*Ms_1492* and Δ*Ms_1492*::pNIT_*Rv0737* strains by fluorescence spectroscopy. Results indicated NR (Figure 5, left) and EB (Figure 5, right) accumulation was higher levels in Δ*Ms_1492* compared to WT. The increased accumulation of both hydrophilic (EB) and lipophilic (NR) dyes indicates a broadly altered membrane architecture, which could impede INH uptake or alter its intracellular activation milieu.

### Rv0737 targeted regulating sigL-rslA operon

**FIGURE 6:**
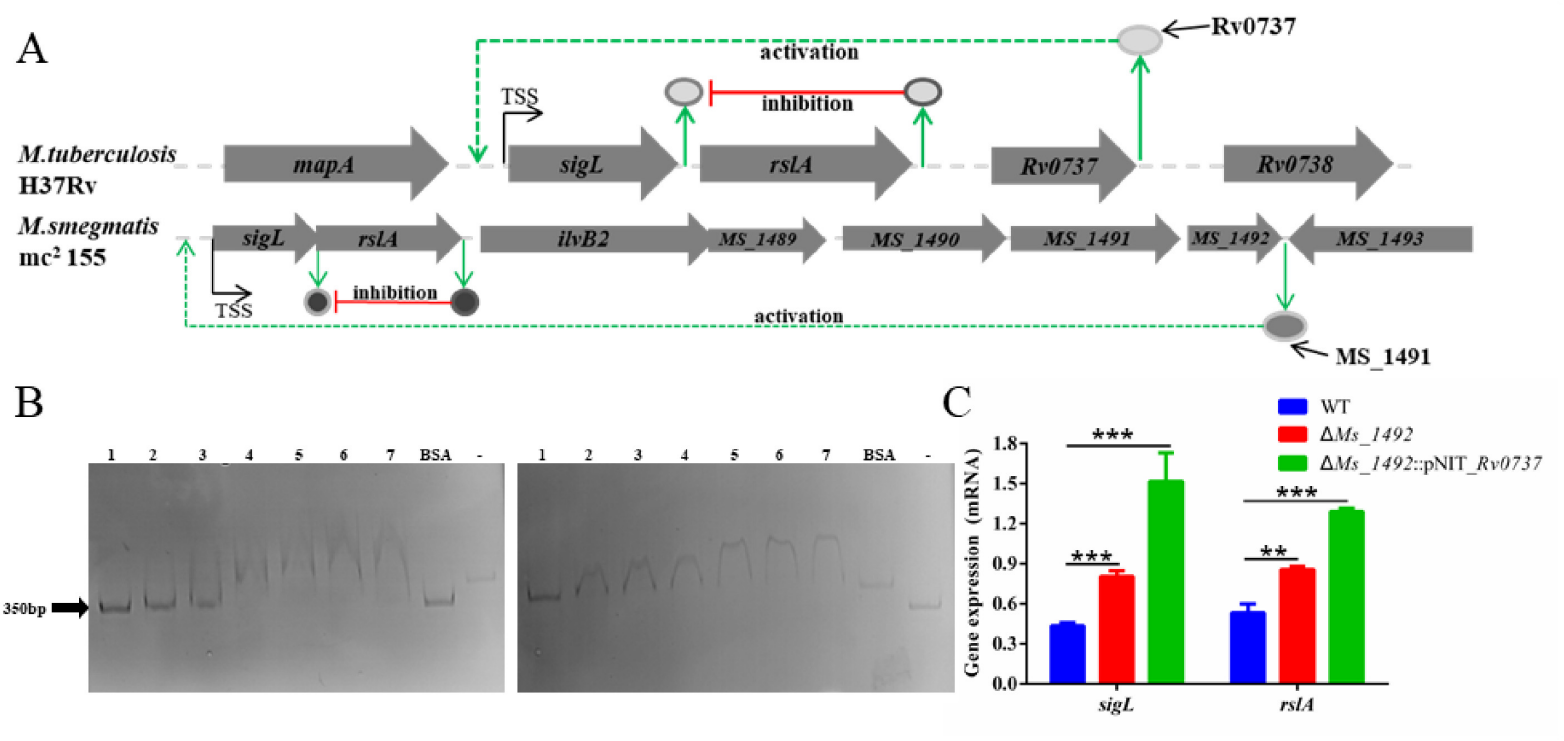
*M. tuberculosis* Rv0737 and *M. smegmatis* homolog Ms_1492 bind to the sigL-rslA operon promoter fragment. **(A)** Approximate mode of regulation **(B)** Rv0737 (left) and Ms_1492 (right) could bind to the sigL-rslA operon promoter fragment. 1–7: The protein concentration was 0, 1, 2, 3, 4, 5 and 6 μM, DNA reaction concentration was 10 ng/mL, and 6 μM BSA was used as the control. “-” is the gene control (left: *Ms_1488* promoter fragment, right: *Ms_1494* promoter fragment). **(C)** Transcription of *sigL* and *rslA* was analyzed using RT-qPCR in the *M. smegmatis* WT, Δ*Ms_1492* and Δ*Ms_1492*::pNIT_*Rv0737* strains. Error bars indicate the standard deviation (SD) and the statistics was calculated using a Student’s unpaired two-tailed *t*-test by the GraphPad Prism software. Symbols are: **p ≤ 0.01, ***p ≤ 0.001.

MarR family genes such as *Rv0880, Rv1404, Rv2887*, regulated target genes adjacent in chromosome (Healy et al., 2016), so we explored the possible target genes near *rv0737* through the mycobrowser website, and then found *sigL* that is a sigma factor. *sigL* and *rslA*, an anti-sigL factor, form an operon (Hahn et al., 2005). We confirmed that the transcriptional regulator *M. tuberculosis* Rv0737 and homolog *M. smegmatis* Ms_1492 regulated sigl-rslA operon directly by binding to its promoter region *in vitro* (Figure 6B). RT-qPCR results indicated that *rv0737* and *Ms_1492* can positively regulate *sigL-rslA* operon expression (Figure 6C). The (Figure 6A and Supplementary Figures 5, 6) described approximate mode of regulation.

### Rv0737 regulates lipid metabolism in response to oxidative and antibiotic stress

**FIGURE 7:**
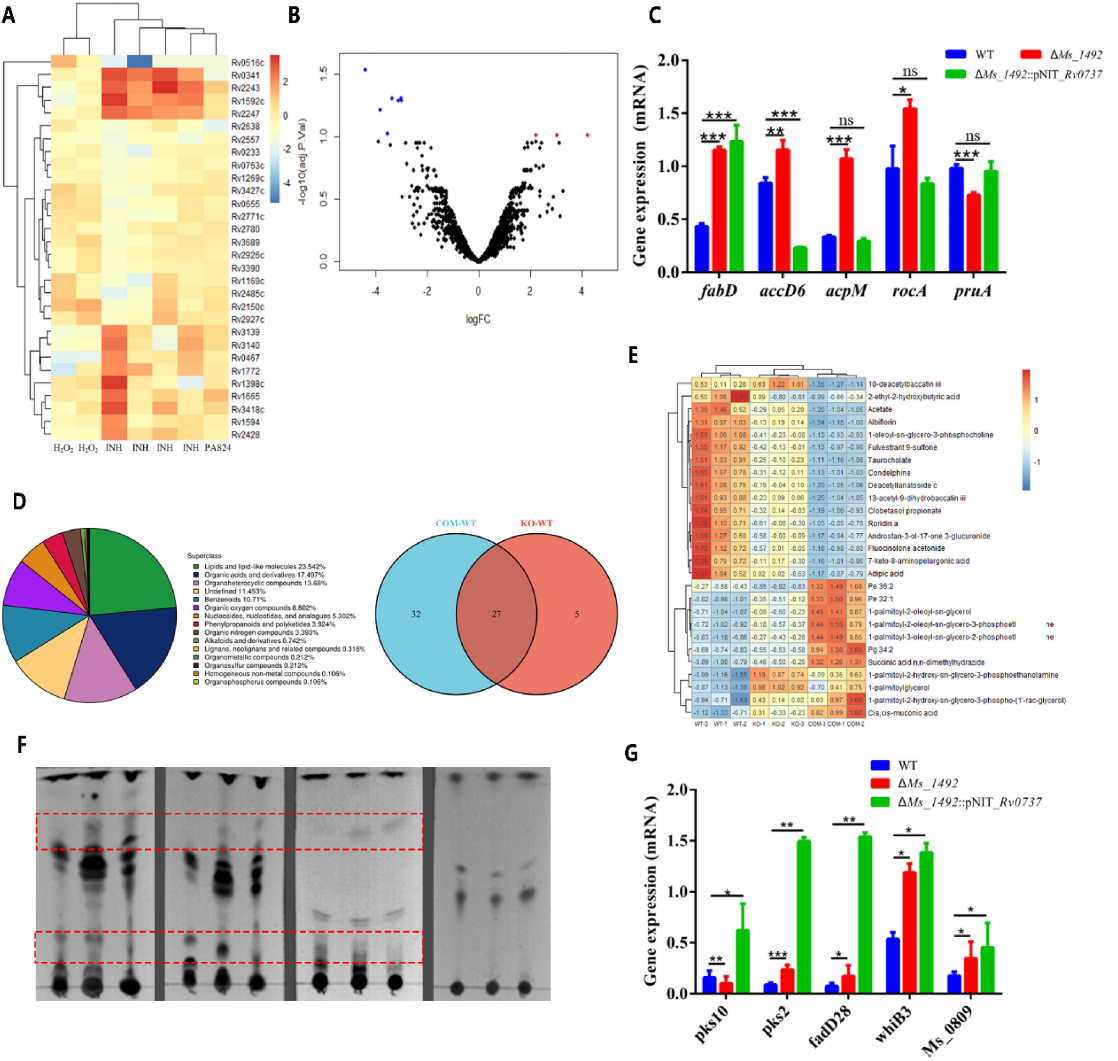
Integrated multi-omics analysis reveals that Rv0737/Ms_1492 remodels lipid metabolism and stress-responsive gene expression. **(A)** Heat map of differential genes drawn from GEO data analysis of *Mycobacterium tuberculosis* treated with H_2_O_2_, INH and PA824. **(B)** Volcano plot of differential gene expression. **(C)** RT-qPCR validation for differential gene. **(D)** Proportion of each superclass metabolites and venn diagrams. **(E)** Differential heatmaps of lipid components. **(F)** Bacterial lipids were analyzed by TLC. The deletion strain has a higher abundance of cell wall lipids than the WT strain. **(G)** Quantitative real-time PCR assays for differential expression of the target genes in WT, ΔMs_1492 and ΔMs_1492::Rv0737 strains. Relative expression levels of the genes were normalized using the 16S rRNA gene as an reference gene. Error bars indicate the standard deviation (SD) and the statistics was calculated using a Student’s unpaired two-tailed *t*-test by the GraphPad Prism software. Symbols are: *^ns^p* > 0.05; *p < 0.05, **p ≤ 0.01, ***p ≤ 0.001.

Given the differential susceptibility of the Δ*Ms_1492* and complemented strains to ROS and isoniazid (INH) stress, we investigated the underlying regulatory mechanism. Analysis of public transcriptomic data revealed that several lipid synthesis genes, along with monooxygenase, anti-sigma factor, and efflux pump genes, were significantly downregulated in *M. tuberculosis* under both H_2_O_2_ and INH stress, suggesting these pathways are targeted by these stressors (Figures 7A, B). Notably, RT-qPCR analysis indicated that the expression changes of these differentially expressed genes in the Δ*Ms_1492* mutant were often different to those observed in the wild-type strain under stress (Figure 7C and Supplementary Figure 7).

To further define the pleiotropic effects of Rv0737 on lipid metabolism, we performed metabolomic analysis. Compared to the wild-type, both the Δ*Ms_1492* and complemented (Δ*Ms_1492*::pNIT_*Rv0737*) strains showed significant alterations in seventeen subclasses of polar lipids (Figures 7D, E). The changed lipid profile itself (Figures 5C, 7D) could modify the environment for INH’s primary target (InhA) or its activation pathway. Thin-layer chromatography (TLC) analysis of total, polar, and non-polar lipid extracts confirmed distinct polar lipid profiles in the mutant and complemented strains compared to the wild-type (Figure 7F). Consistent with this, RT-qPCR assays demonstrated that the deletion of *Ms_1492* led to the upregulation of lipid synthesis genes *pks2*, *fadD28*, and *Ms_0809*, with *whiB3* being highly significantly upregulated. This upregulation was even more pronounced in the complemented strain (Figure 7G). The upregulation of *pks2* (sulfolipid synthesis) and *fadD28* (fatty acyl-AMP ligase) in the complemented strain (Figure 7G) aligns with the observed shifts in specific polar lipid subclasses like glycerophospholipids and fatty acid conjugates (Table 1). As *M. smegmatis* lacks virulence lipids like PDIM, these genes are likely involved in the synthesis of non-toxic lipids in this model organism. The significant upregulation of the redox regulator *whiB3* correlates directly with the altered oxidative stress response phenotypes. The reduction in metabolites like 7-keto-8-aminopelargonic acid (a biotin/FA synthesis precursor) in the growth-impaired complemented strain provides a metabolic explanation for the observed growth defect. Collectively, these results demonstrate that the MarR transcription factor Rv0737 plays a critical role in regulating bacterial lipid synthesis in response to stress.

**TABLE 1:** Differential lipid species identified in WT vs. Δ*Ms*_*1492*/complemented strain by untargeted lipidomics.

Name	Adduct	m.z	Class	Sub class
(3,5,9,13,16,17,22r,23r,24r)-3-{[-l-arabinofuranosyl-(1- > 2)-[-d-glucopyranosyl-(1- > 3)]–l-arabinopyranosyl]oxy}-20-hydroxy-24-methyl-16,23:16,30-diepoxydammar-25-en-22-yl acetate (abbreviation: Acetate)	[M+K]^+^	1009.482	Prenol lipids	Triterpenoids
1-oleoyl-sn-glycero-3-phosphocholine	[M+K]^+^	560.3272	Glycerophospholipids	Glycerophosphocholines
1-palmitoyl-2-hydroxy-sn-glycero-3-phospho-(1’-rac-glycerol)	[M−H]^–^	483.2734	Glycerophospholipids	Glycerophosphoglycerols
1-palmitoyl-2-hydroxy-sn-glycero-3-phosphoethanolamine	[M−H]^–^	452.2786	Glycerophospholipids	Glycerophosphoethanolamines
1-palmitoyl-2-oleoyl-sn-glycero-3-phosphoethanolamine	[M+K]^+^	718.5377	Glycerophospholipids	Glycerophosphoethanolamines
1-palmitoyl-2-oleoyl-sn-glycerol	[M+H−H_2_O]^+^	577.5187	Glycerolipids	Diradylglycerols
1-palmitoyl-3-oleoyl-sn-glycero-2-phosphoethanolamine	[M−H]^–^	716.5242	Glycerophospholipids	Glycerophosphates
1-palmitoylglycerol	[M+H−H_2_O]^+^	313.2737	Glycerolipids	Monoradylglycerols
10-deacetylbaccatin iii	[M+H−2H_2_O]^+^	509.2201	Prenol lipids	Diterpenoids
13-acetyl-9-dihydrobaccatin iii	[M+Na]^+^	653.2414	Prenol lipids	Diterpenoids
2-ethyl-2-hydroxybutyric acid	[M−H]^–^	131.0715	Fatty acyls	Fatty acids and conjugates
7-keto-8-aminopelargonic acid	[M+H]^+^	188.1281	Fatty acyls	Fatty acids and conjugates
Adipic acid	[M−H]^–^	145.0508	Fatty acyls	Fatty acids and conjugates
Albiflorin	[M+H−C_6_H_10_O_5_]^+^	319.1363	Prenol lipids	Terpene glycosides
Androstan-3-ol-17-one 3-glucuronide	[M−H+2Na]^+^	511.2151	Steroids and steroid derivatives	Steroidal glycosides
*Cis,cis*-muconic acid	[M^–^H]^–^	141.0171	Fatty acyls	Fatty acids and conjugates
Clobetasol propionate	[M+H]^+^	467.1889	Steroids and steroid derivatives	Pregnane steroids
Condelphine	[M+Na]^+^	472.275	Prenol lipids	Diterpenoids
Deacetyl-lanatoside C	[M+Na]^+^	965.4561	Steroids and steroid derivatives	Steroid lactones
Fluocinolone acetonide	[M+H]^+^	495.241	Steroids and steroid derivatives	Pregnane steroids
Fulvestrant 9-sulfone	[M+Cl]^–^	657.2982	Steroids and steroid derivatives	Estrane steroids
Pe 32:1	[M-H]^–^	688.4927	Glycerophospholipids	Glycerophosphoethanolamines
Pe 36:2	[M-H]^–^	742.5403	Glycerophospholipids	Glycerophosphoethanolamines
Pg 34:2	[M-H]^–^	745.5033	Glycerophospholipids	Glycerophosphoglycerols
Roridin A	[M+Na]^+^	555.2412	Prenol lipids	Sesquiterpenoids
Succinic acid n,n-dimethylhydrazide	[M+H−H_2_O]^+^	143.0815	Fatty acyls	Fatty acids and conjugates
Taurocholate	[M+H]^+^	516.301	Steroids and steroid derivatives	Bile acids, alcohols and derivatives

## Discussion

Mycobacteria have a complex regulatory network that includes two-component regulatory systems, serine/threonine protein kinases, sigma factors, whiB-like proteins and MarR family transcription factors. These kinases and regulatory factors interact with each other to target different functional factors of bacteria and regulate many pathways such as mycobacterial growth metabolism, cell division, nutrient deficiency, lipid synthesis, antibiotic resistance and stress sensing (Gong et al., 2019; Burian et al., 2012; Parish, 2014; Prisic and Husson, 2014; Zhou et al., 2018). Among them, the regulatory function of the mycobacterial MarR family transcription factors are still in the preliminary exploration stage. A total of 9 MarR family transcription factors were identified and annotated in *M. tuberculosis*: Rv0042c, Rv0737, Rv0880, Rv1049, Rv1404, Rv2011c, Rv2327, Rv2887, and Rv0678 (Gong et al., 2019; Radhakrishnan et al., 2014). Our study found that overexpression of the MarR family transcription factor Rv0737 delayed bacterial growth and led to bacterial lengthening and reduced biofilm formation. These phenomena may be related to the bacterial metabolic pathways that regulated by the sigL-rslA operon. Rv0737, via sigL-rslA, modulates lipid synthesis pathways critical for maintaining cell envelope properties necessary for biofilm formation under stress.

DNA-binding motifs of MarR family transcription factors are highly conserved, and the location of their cis-regulatory elements determines whether transcription factors function as activators or repressors, and mostly function as transcriptional repressors (Radhakrishnan et al., 2014). In terms of gene transcription activation, it is mainly found that OhrR, a member of the MarR family of Streptomyces coelicolor, binds to the -35 region of its adjacent gene ohrA and recruits RNA polymerase to activate transcription (Oh et al., 2007).

SigL interacts with the anti-SigL factor RslA that inactivated SigL. SigL, as a less-studied gene among the 13 sigma factors, encodes the recognition subunit of RNA polymerase. At present, it is mainly found that it participates in the regulation of lipid synthesis, redox stress and DNA repair reactions by targeting genes such as pks7-pks10, Rv1138c-Rv1139c and recN (Hahn et al., 2005). Through the results of RT-qPCR experiments, we found that when Rv0737 was overexpressed, not only the mRNA level of pks10 was increased, but also pks2 was increased (involved in sulfolipid -1 synthesis) and fadD28 (involved in phthiocerol dimycocerosates (PDIM) synthesis) increased dramatically (Goyal et al., 2006; Sirakova et al., 2001).

However, virulence lipids such as PDIM were not found in *M. smegmatis*, which may be due to the lack of related virulence lipid synthesis cofactors in non-pathogenic *M. smegmatis* (Goyal et al., 2006). However, the high expression of these genes may lead to block the synthesis of other lipids, and synthesize some lipid molecules with carbon chains of different lengths and functional groups, thereby affecting the lipid composition and structural changes of the cell wall. The research results of this subject also showed that the cell wall permeability of the Δ*Ms_1492* strain was enhanced, and the ATP content was increased, which may be due to the change of lipid composition leading to the enhancement of related metabolic pathways and the increase of energy synthesis. Based on the metabolomics data, seventeen subclass of the polar lipid (triterpenoids, glycerophosphocholines, glycerophosphoglycerols, glycerophosphoethanolamines, diradylglycerols, glycerophosphates, monoradylglycerols, diterpenoids, fatty acids and conjugates, terpene glycosides, steroidal glycosides, pregnane steroids, steroid lactones, estrane steroids, sesquiterpenoids, bile acids, and alcohols and derivatives) shows different contents in WT, Δ*Ms_1492* and Δ*Ms_1492*::pNIT_*Rv0737* strains. Especially, 7-keto-8-aminopelargonic acid, a pyridoxal 5’-phosphate-dependent enzyme, catalyzes the decarboxylative condensation of l-alanine with pimeloyl-CoA to form coenzyme A in the first committed step of biotin biosynthesis. It is the first step in fatty-acid biosynthesis which is essential for the growth and development of most organisms. The lipid was significantly reduced in Δ*Ms_1492*::pNIT_*Rv0737* strain that hinders the cell division process and reduces the growth rate. The pronounced cell elongation observed upon Rv0737 overexpression (Figures 2F, 4F, G) points to a disruption in the cell division machinery. While we found a corresponding decrease in ftsZ mRNA (Figure 4H), suggesting that dysregulation of this key division protein may contribute to the phenotype, this remains a correlative observation. The division defect could also arise from indirect effects, such as altered membrane fluidity, energy status, or the expression of other division-related genes. Therefore, establishing a direct causal link between Rv0737 activity and FtsZ function will require further investigation, including analysis of FtsZ protein levels and localization, visualization of division septa by microscopy, and genetic complementation experiments to determine whether restoring *ftsZ* expression can rescue the division phenotype.

While the non-pathogenic model *M. smegmatis* has been instrumental in enabling the genetic and mechanistic dissection reported here, we acknowledge that it lacks the full repertoire of virulence lipids, such as PDIMs, that characterize *M. tuberculosis* pathogenesis. Therefore, direct extrapolation of the specific lipid compositional changes to the context of human infection warrants caution. However, our study was designed to elucidate the core, conserved regulatory logic of the Rv0737/Ms_1492 transcription factor. The successful cross-species complementation—where the *M. tuberculosis* protein restored function in the *M. smegmatis* mutant—provides compelling genetic evidence that this regulatory node and its functional output are fundamentally conserved. The pathway we have identified, wherein Rv0737/Ms_1492 targets the sigL-rslA operon to modulate cell envelope integrity and stress adaptation, represents a highly promising and novel regulatory axis in mycobacterial physiology. Consequently, the logical and crucial next step will be to validate the role of this pathway in *M. tuberculosis* itself, particularly its impact on the synthesis of virulence-associated lipids and its contribution to pathogenesis and drug tolerance in the human pathogen. This work establishes a solid foundation and a clear mechanistic framework for those essential future investigations. Future studies aimed at determining the protein’s structure, mapping its precise DNA-binding site, and testing the effect of oxidants or specific ligands on its activity *in vitro* and *in vivo* will be essential to fully elucidate the molecular switch that controls this regulatory pathway.

The enormous adaptability of microorganisms is the key to their competition in nature, but also poses a challenge to the antibiotic treatment of human diseases (Stubbendieck and Straight, 2016). During infection of a specific host, bacteria reprogram gene expression through a wide variety of transcription factors in response to a range of different stresses (Zhou et al., 2018). The MarR family proteins are sensors of environmental stress, virulence, and catabolism of aromatic compounds, particularly in adapting bacteria to environmental stresses such as antibiotics, toxic chemicals, and ROS. The regulatory responses involved in MarR family transcription factors are mainly divided into three categories, including (1) environmental stress responses (antibiotics, disinfectants, etc.), (2) regulation of virulence genes (virulence lipids, secreted antigens, etc.), and (3) degradation of lipid-soluble (usually aromatic) compounds(Alekshun and Levy, 1997; Gong et al., 2019).

**FIGURE 8:**
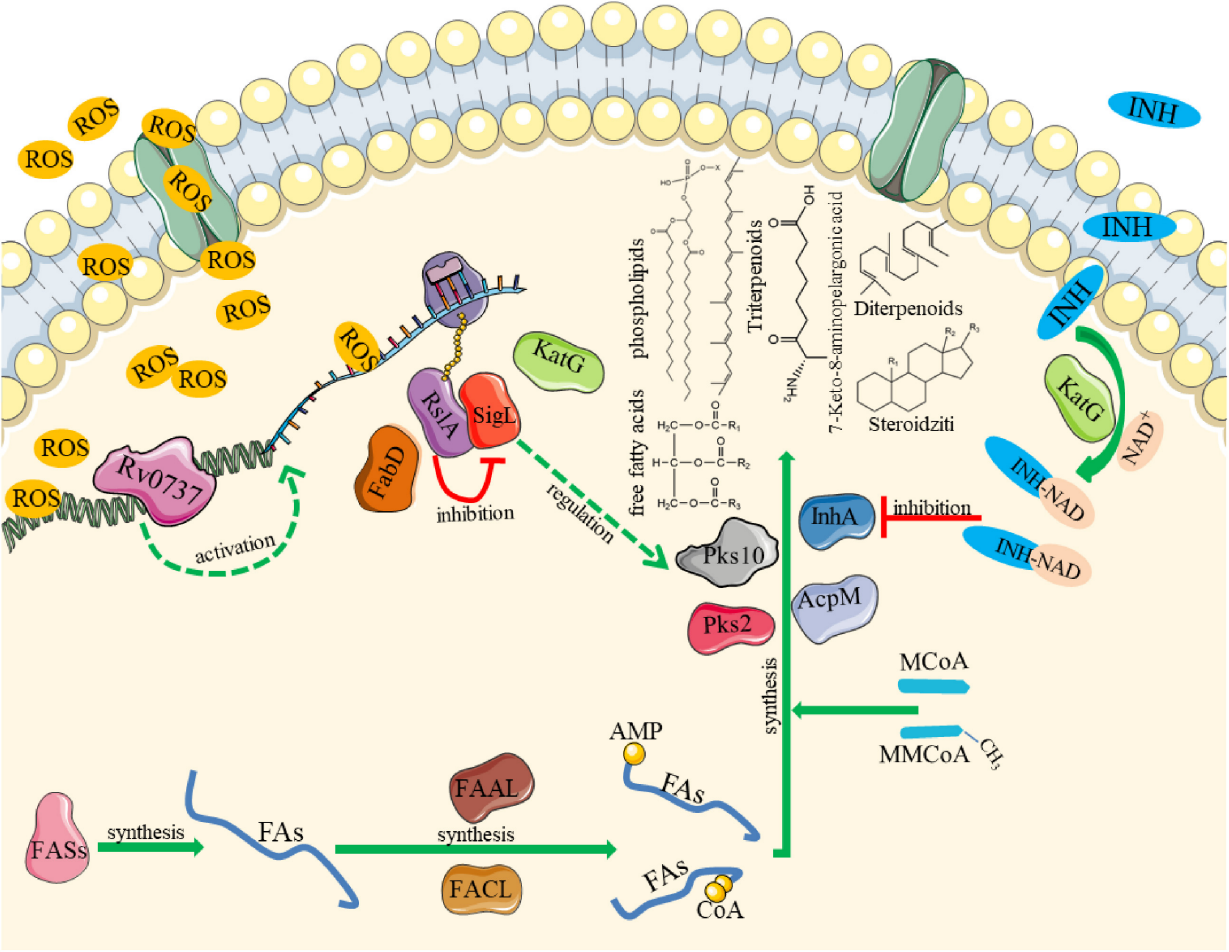
Diagram of the regulatory mechanism of Rv0737. Rv0737 affects bacterial growth and lipid synthesis-related pathways by targeting the sigL-rslA operon to regulate the expression of lipid synthesis genes such as acpM, pks2/10 and katG. FA, fatty acid; FASs, fatty acid synthase; FAAL, fatty acyl-AMP ligase; FACL, fatty acyl-CoA ligase; MCoA, malonyl CoA.

In order to further explore the effect of Rv0737 on bacterial cell wall lipid synthesis and oxidative response, we treated it with H_2_O_2_ and isoniazid. We found that the apoplectic strains showed a sensitive phenotype, which may be that the lipid synthesis pathway is targeted blocking by INH. Resulting in structural changes in the *M. smegmatis* cell wall and becomes vulnerable to damage. The Δ*Ms_1492* strain showed mild tolerance to oxidative stress and isoniazid, and its cell membrane permeability was altered. This indicates that the lipid composition of Δ*Ms_1492* strain is more favorable for bacterial survival than the Δ*Ms_1492*::pNIT_*Rv0737* strain. The experimental data show that the overexpression of Rv0737 may affect the growth and division of bacteria, and the detection results of ROS efflux capacity and ATP content indicate that Rv0737 affects the oxidative response capacity of bacteria, dynamically regulates the expression of target genes, and enhances the resistance of bacteria to environmental stress. Seventeen subclass of the polar lipid (triterpenoids, glycerophosphocholines, glycerophosphoglycerols, glycerophosphoethanolamines, diradylglycerols, glycerophosphates, monoradylglycerols, diterpenoids, fatty acids and conjugates, terpene glycosides, steroidal glycosides, pregnane steroids, steroid lactones, estrane steroids, sesquiterpenoids, bile acids and alcohols and derivatives) shows different contents in WT, Δ*Ms_1492* and Δ*Ms_1492*::pNIT_*Rv0737* strains. In summary, we characterized the *M. tuberculosis* Rv0737 targeting the sigL-rslA operon involved in the regulation of bacterial growth and division, cell membrane permeability and ATP synthesis, and affecting cell wall lipid synthesis, oxidative response and INH tolerance (Figure 8).

## Data Availability

The original contributions presented in this study are included in this article/Supplementary material, further inquiries can be directed to the corresponding authors.
