# Supplementary figures and images for: Mycobacterium tuberculosis MarR family transcription factor Rv0737 regulates bacterial growth and lipid synthesis by targeting the sigL-rslA operon
Source: Front Microbiol. 2026 Mar 6;17:1727573. doi: 10.3389/fmicb.2026.1727573 (PMC13002591; doi:10.3389/fmicb.2026.1727573)

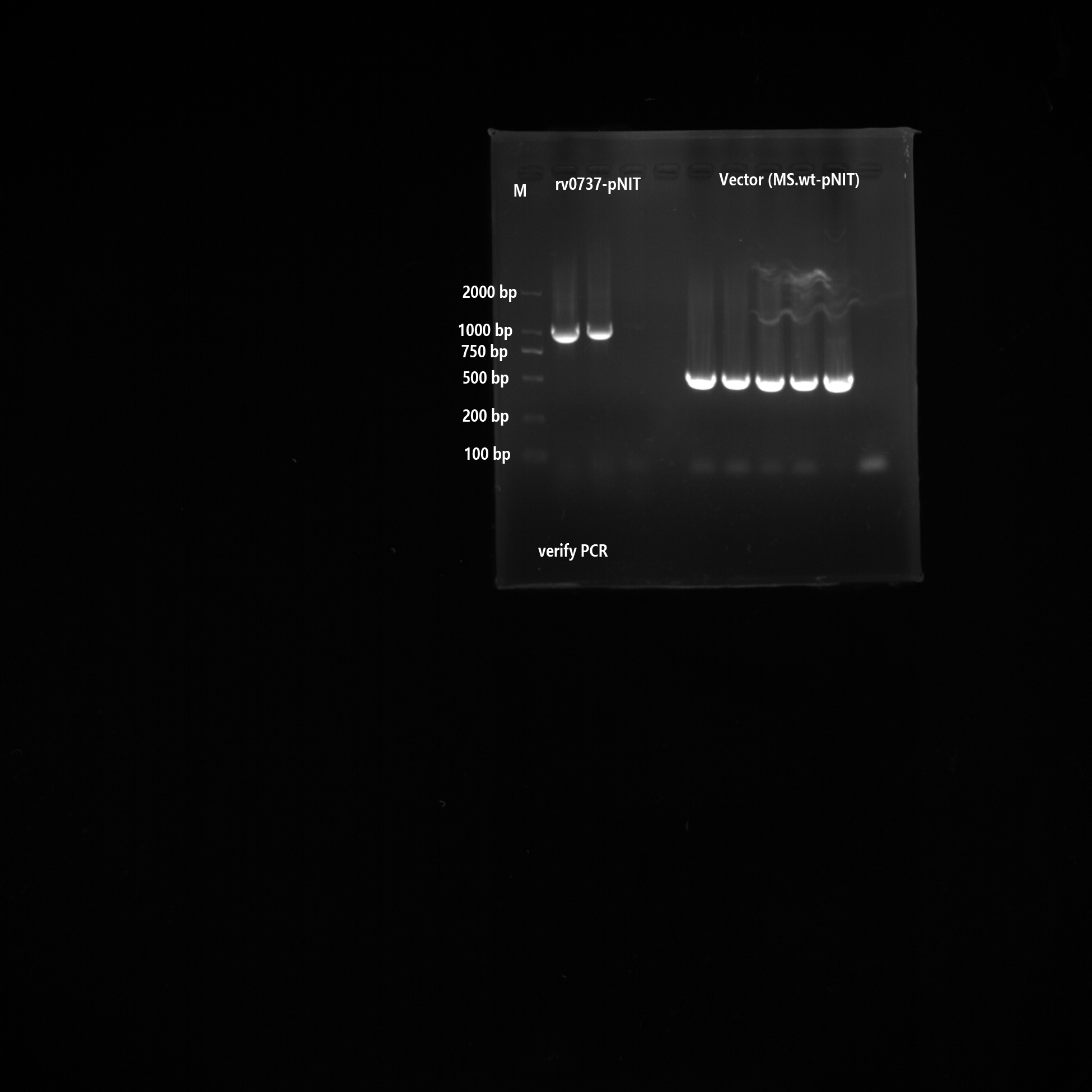

Supplement: Supplementary file 1 [file Image_1.tif]

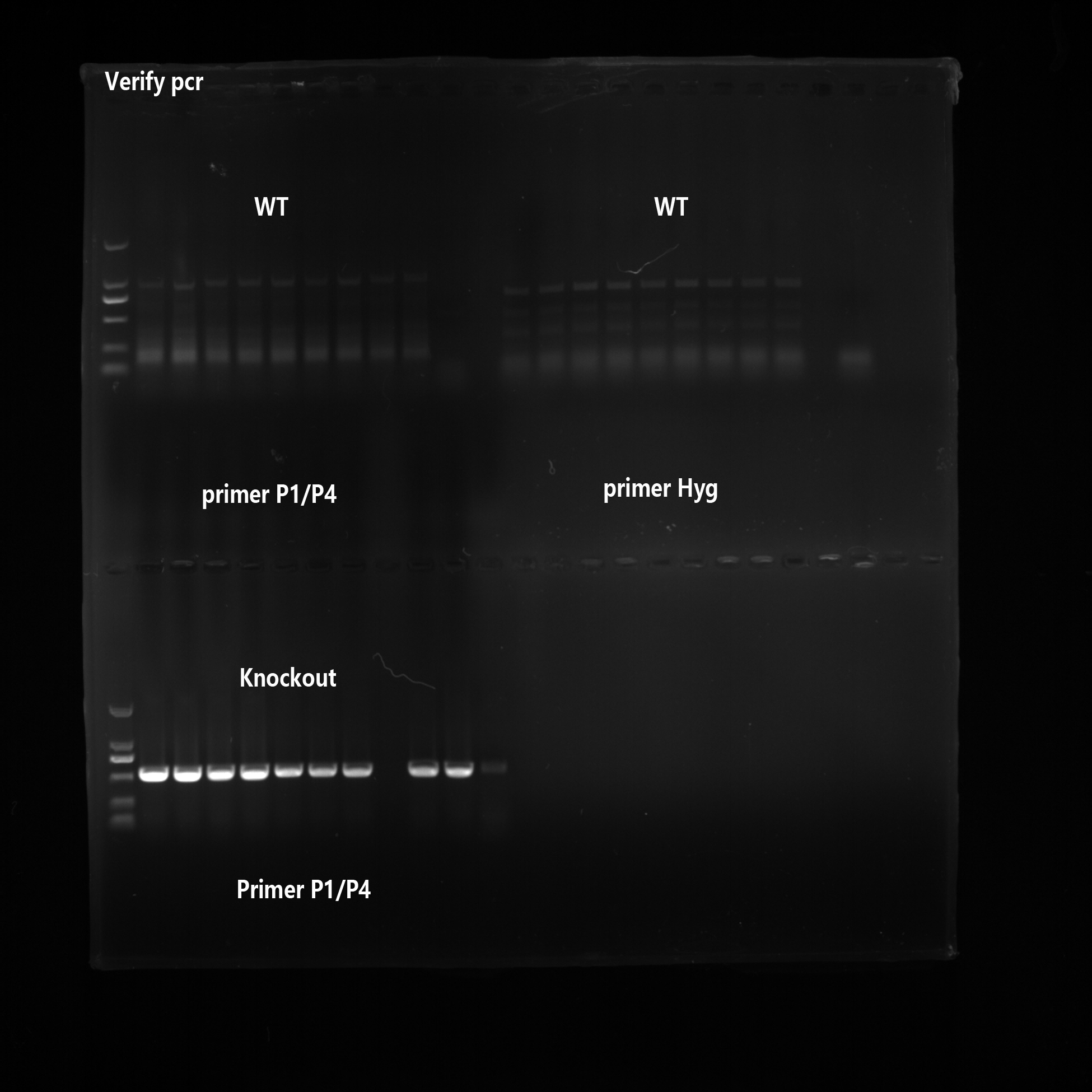

Supplement: Supplementary file 2 [file Image_2.tif]

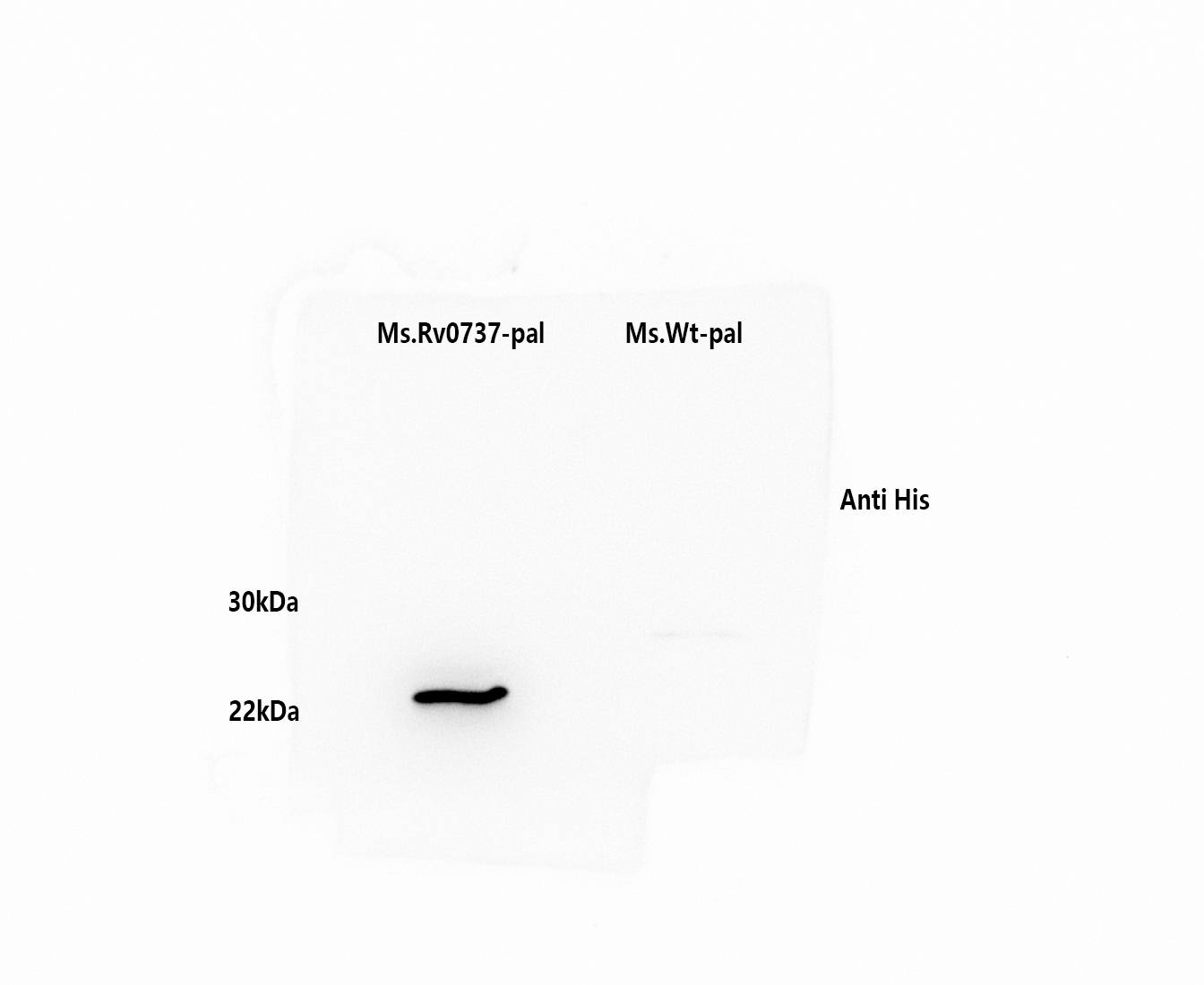

Supplement: Supplementary file 3 [file Image_3.tif]

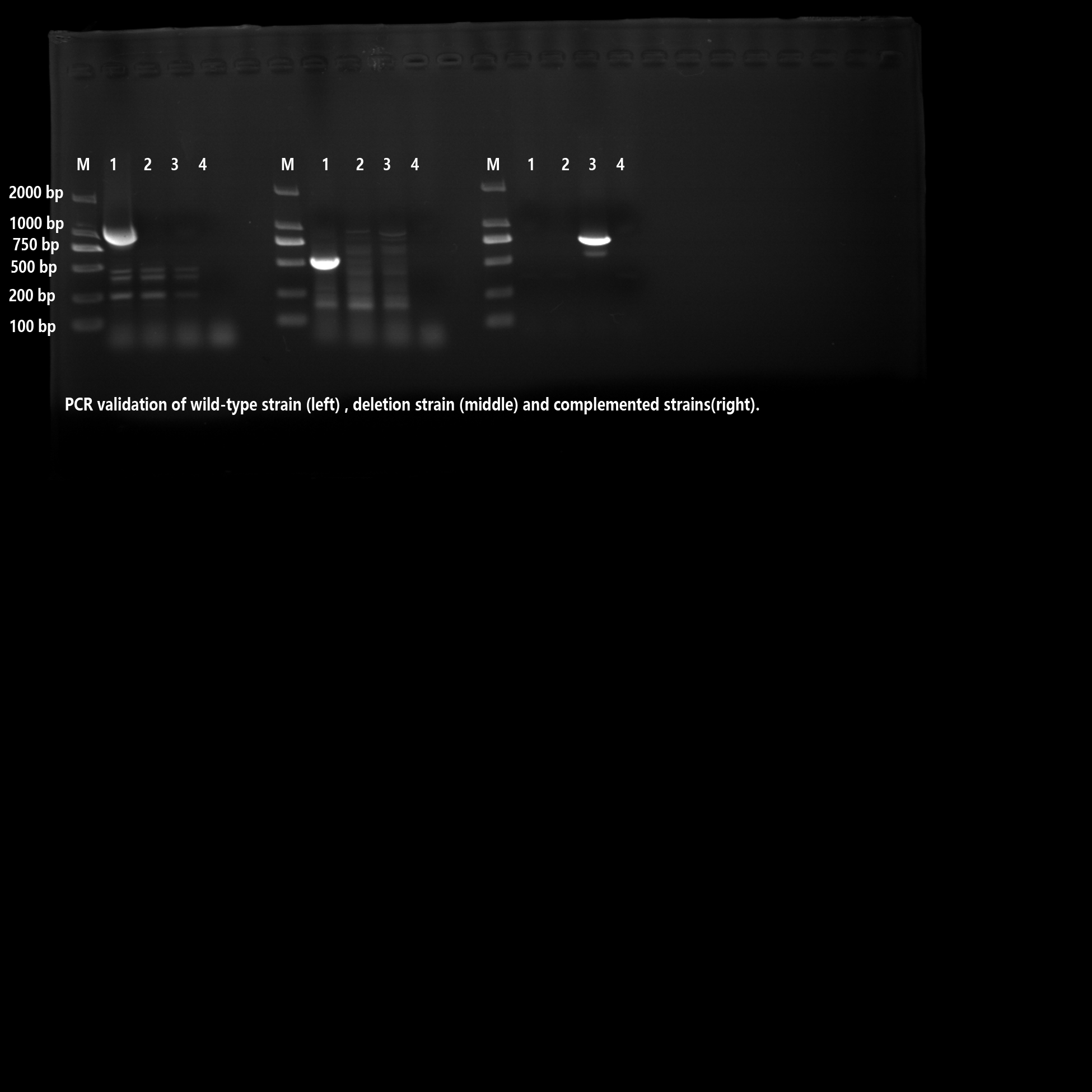

Supplement: Supplementary file 4 [file Image_4.tif]

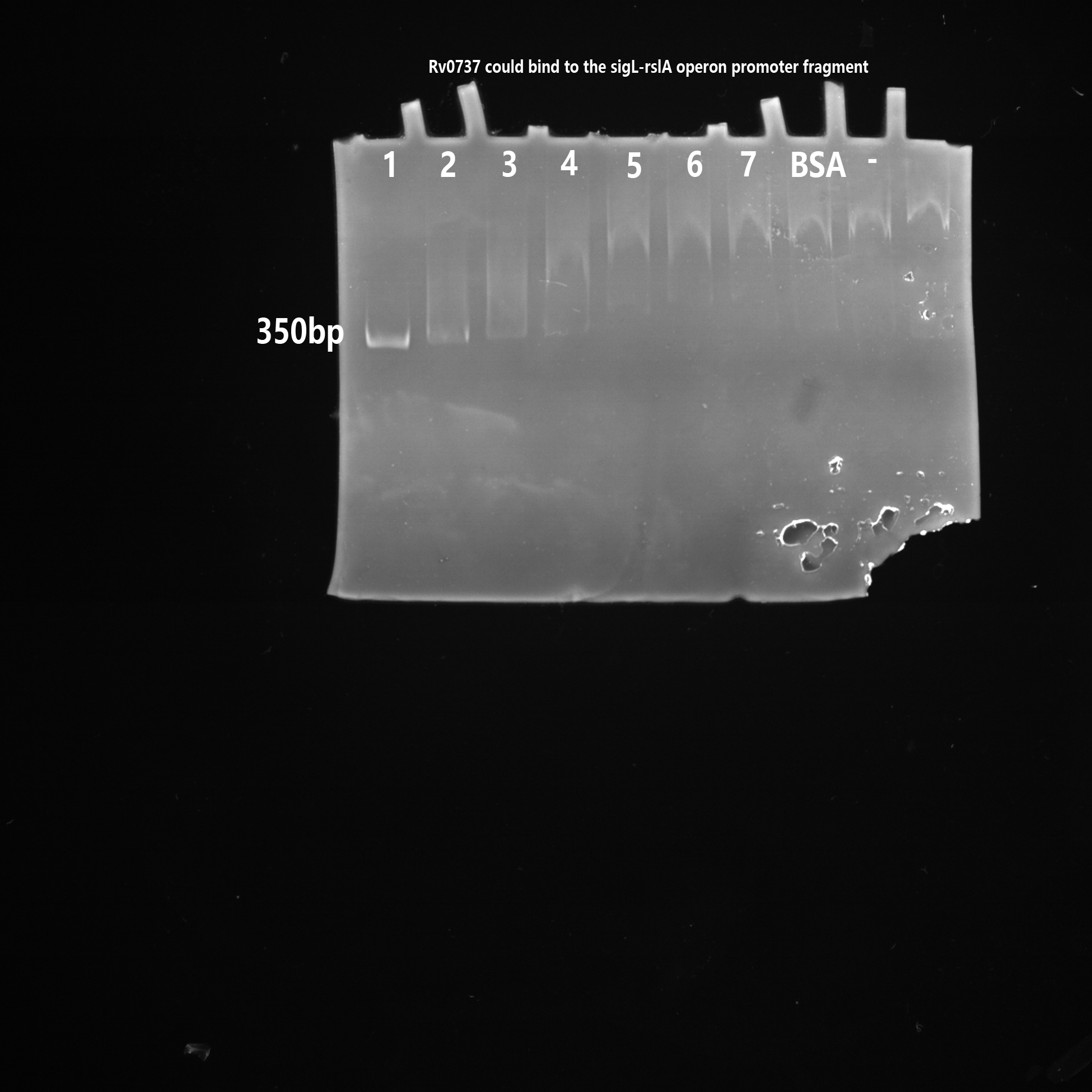

Supplement: Supplementary file 5 [file Image_5.tif]

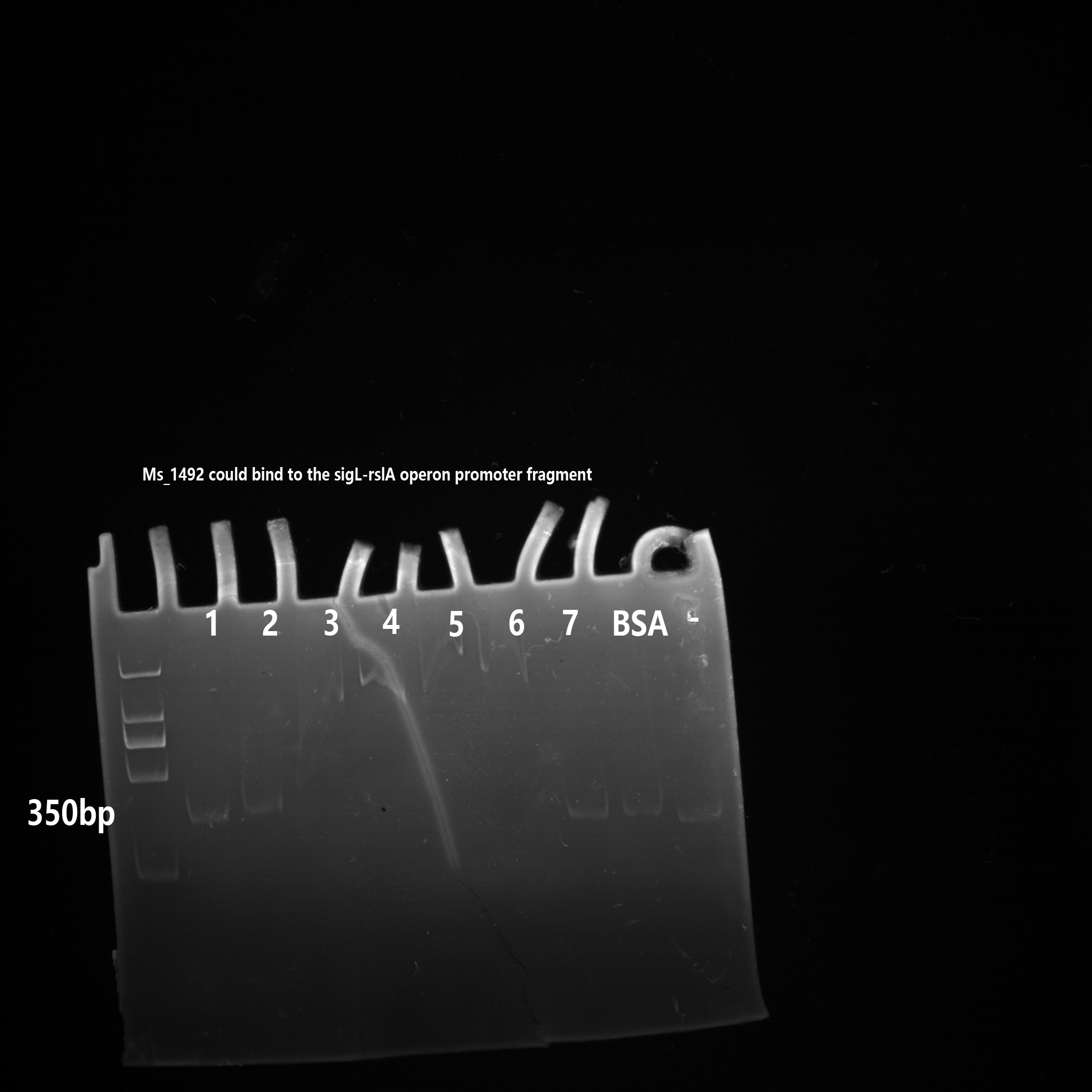

Supplement: Supplementary file 6 [file Image_6.tif]

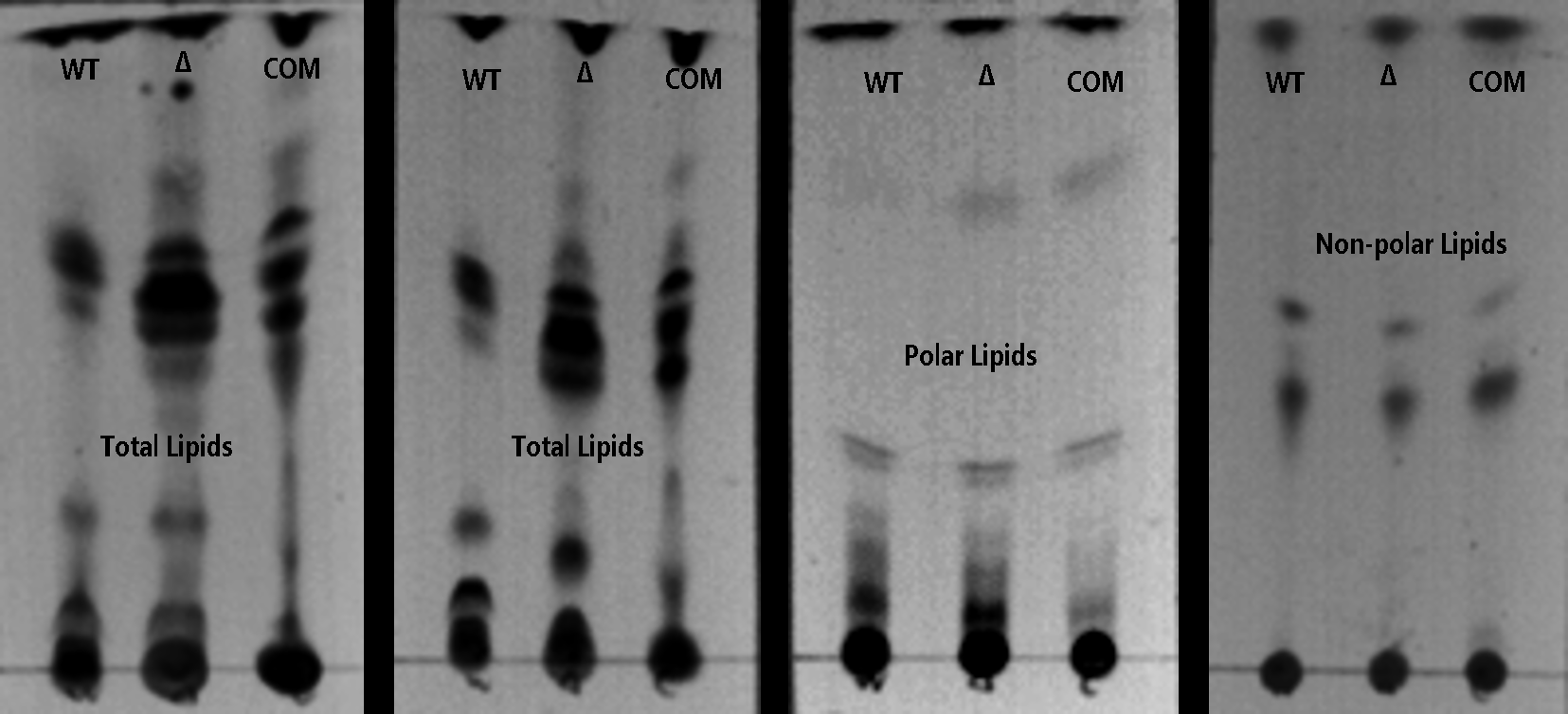

Supplement: Supplementary file 7 [file Image_7.png]

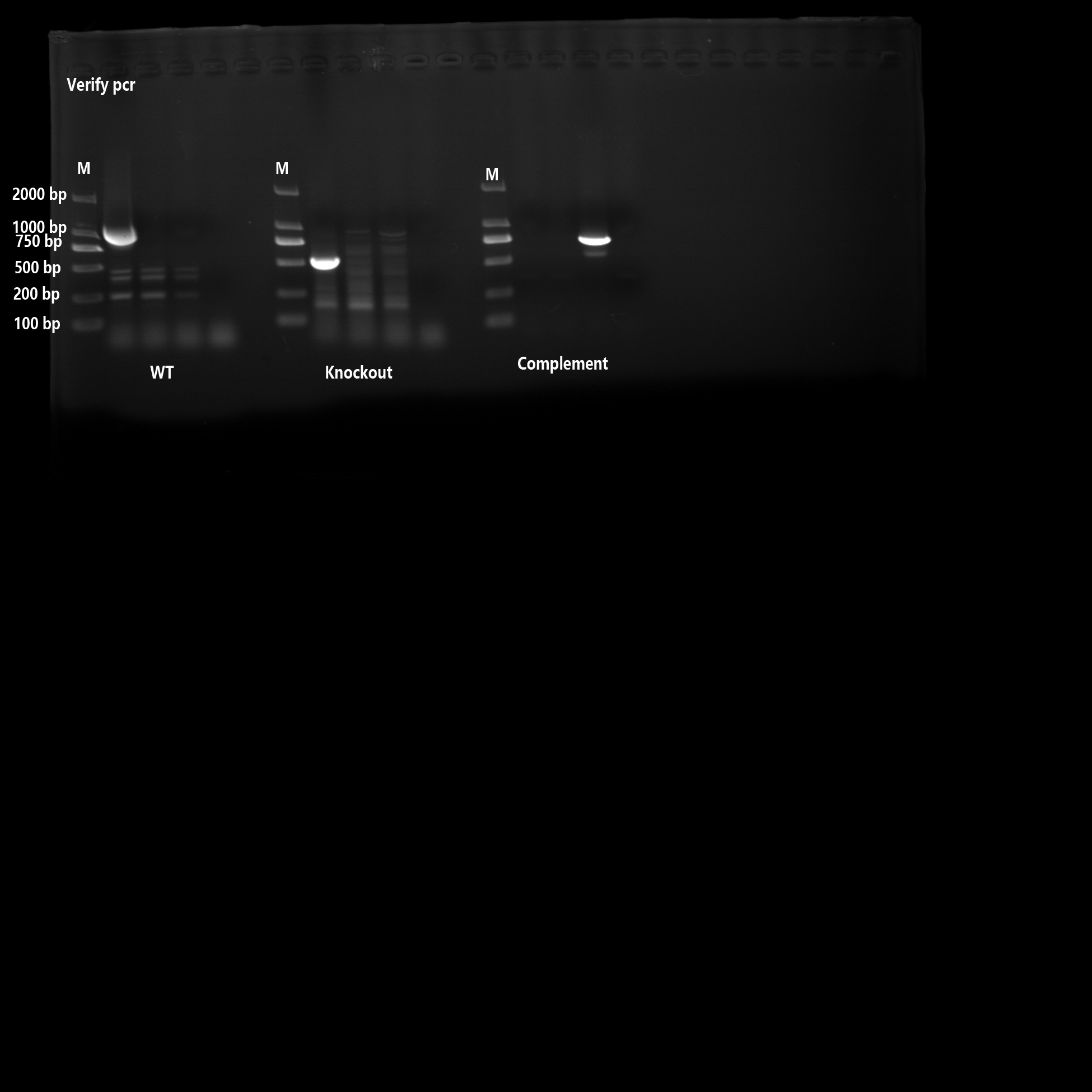

Supplement: Supplementary file 8 [file Image_8.tif]
